# Vaccine‐Induced Immune Thrombotic Thrombocytopenia Caused by Zoetis PR‐VAC PLUS in Pseudorabies Virus‐Free Danish Purebred Pigs

**DOI:** 10.1002/vms3.70222

**Published:** 2025-01-27

**Authors:** Chien‐Cheng Chen

**Affiliations:** ^1^ Department of Veterinary Medicine National Chiayi University Chiayi City Taiwan

**Keywords:** gastric ulcer, haemorrhagic bowel syndrome, osteochondrosis, PRV vaccine, vaccine‐induced immune thrombotic thrombocytopenia, VITT

## Abstract

This case report highlights a potential vaccine safety concern associated with the Pseudorabies virus (PRV) live vaccine, which warrants further investigation for comprehensive understanding. Vaccine‐induced immune thrombotic thrombocytopenia (VITT), a novel syndrome of adverse events following adenovirus vector COVID‐19 vaccines, was observed after vaccination with Zoetis PR‐VAC PLUS. This led to a 100% morbidity and high mortality among PRV‐free Danish purebred pigs from Danish Genetics Co. Clinical signs and gross presentations due to the post‐thrombotic syndrome (PTS) and pulmonary embolism (PE) included (1) PE causing severe pulmonary oedema, which led to shortness of breath and respiratory failure. PTS causing (2) osteochondrosis (OCD)/leg weakness syndrome; (3) acute gastric ulcers and bleeding; (4) haemorrhagic bowel syndrome; (5) myocardial infarction or heart failure and (6) noticeable varicose veins. (7) Thrombocytopenia along with reddish or purplish spots appeared. Definitive diagnosis was based on the following as human medicine: (1) clinical signs appearing 4–42 days post‐vaccination; (2) thrombocytopenia; (3) presence of thrombus in lung vessels, alveolar septa vessels, gastric vessels and small intestine, as well as other organs, as confirmed by histopathologic examination; (4) positive result from anti‐heparin/PF4 ELISA testing. VITT in pigs, not previously reported, could be attributed to several factors: (1) Differences in diagnosing pigs compared to humans, especially considering that VITT associated with COVID‐19 vaccines has only recently been identified. (2) Challenges in linking clinical signs such as pulmonary oedema, OCD/leg weakness, gastric ulcers and haemorrhagic bowel syndrome to thrombosis. (3) Delayed onset of clinical signs 4–42 days post‐vaccination, unlike common vaccine side effects. (4) Clinical signs were varied and illogical, and ranged from mild to moderate with low mortality if there were no other complications or conditions. (5) Difficulty in diagnosing the condition due to the presence of common pathogens like PRRSV and PCV2. (6) Breeding/genetic factors can be an issue when breeders with strong immunity, who may also be highly sensitive to VITT, are selected. The potential problem of VITT might not be detected because no PR vaccine is used in Denmark.

## Graphical abstract

Vaccine‐induced immune thrombotic thrombocytopenia (VITT), a novel syndrome of adverse events following adenovirus vector COVID‐19 vaccines, was observed after vaccination with Zoetis PR‐VAC PLUS. This resulted in 100% morbidity in PRV‐free Danish pure breeders from Danish Genetics Co., causing severe pulmonary oedema, gastric ulcers, haemorrhagic bowel syndrome, osteochondrosis (OCD)/leg weakness syndrome, varicose veins, thrombocytopenia with reddish or purplish spots and death. Diagnosis based on: (1) symptoms appearing 4–42 days post‐vaccination; (2) thrombocytopenia; (3) presence of thrombus and (4) positive result from anti‐/PF4 ELISA testing.

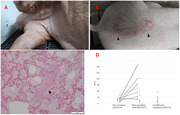

## 1 History and Course of Disease

Our farm is in Tainan City, Taiwan. It was established as a new farm in March 2023. We received two batches of Danish breeders from Danish Genetics Co. The first batch, consisting of 4 boars and 31 gilts of the Landrace and Yorkshire strains, arrived on 11 March 2023, and the second batch, consisting of 16 boars and 81 gilts of the Landrace, Yorkshire and Duroc strains, arrived on 11 September 2023. Zoetis PR‐VAC PLUS, a live vaccine, each dose (2 mL) containing Pseudorabies virus (Bucharest strain) at least 10^4.0^TCID_50_ and Amphigen adjuvant, was administered on all PR vaccine programmes.

**FIGURE 1:**
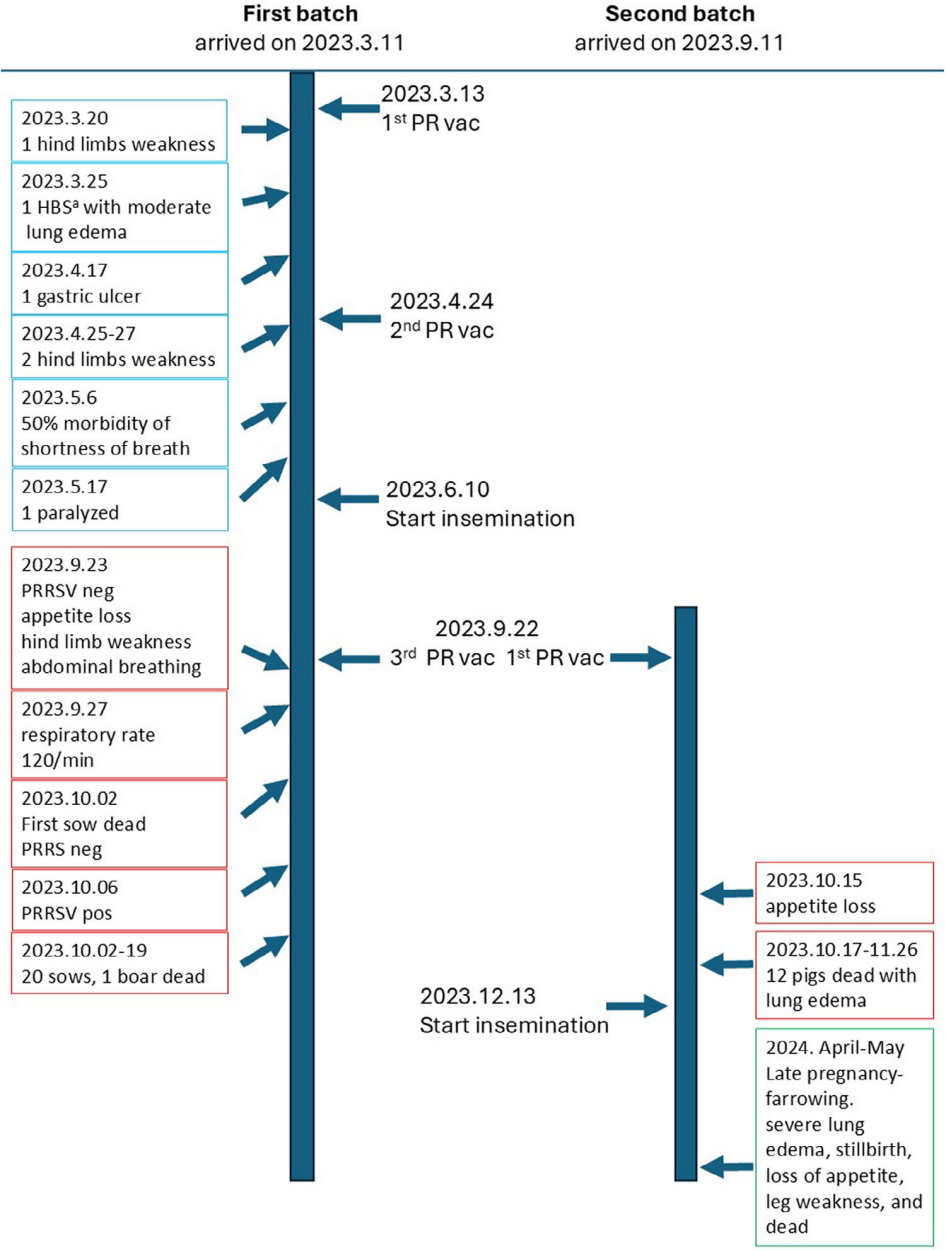
Timeline of events from 11 March 2023 to May 2024, speculatively related to adverse reactions from the PR vaccination. Red blocks indicate events where severe clinical signs led to the discovery of VITT. Blue blocks represent events traced back after diagnosing VITT. Green blocks indicate follow‐up events suggesting that VITT may cause lifelong harm. ^a^Haemorrhagic bowel syndrome.

Figure 1 presents all events from 11 March 2023 to May 2024, which speculatively related to the adverse reactions of PR vaccination, written dispersedly in all chapters. The clinical signs occurred within 1 month after vaccination and during the late pregnancy to farrowing. However, the depiction of clinical signs and diagnosis focuses on events after 22 September, as the third dose for the first batch (10‐month‐old pregnant sows and boars) and as a first‐time vaccination for the second batch (4–5‐month‐old breeders). Severe clinical signs with 100% morbidity and high mortality were found within 50 days post‐vaccination (Figure 1).

Serological testing of porcine reproductive and respiratory syndrome virus (PRRSV) antibody and viremia, and PRV‐gE antibody were all negative on 23 September and before.

## 2 Clinical Signs

**FIGURE 2:**
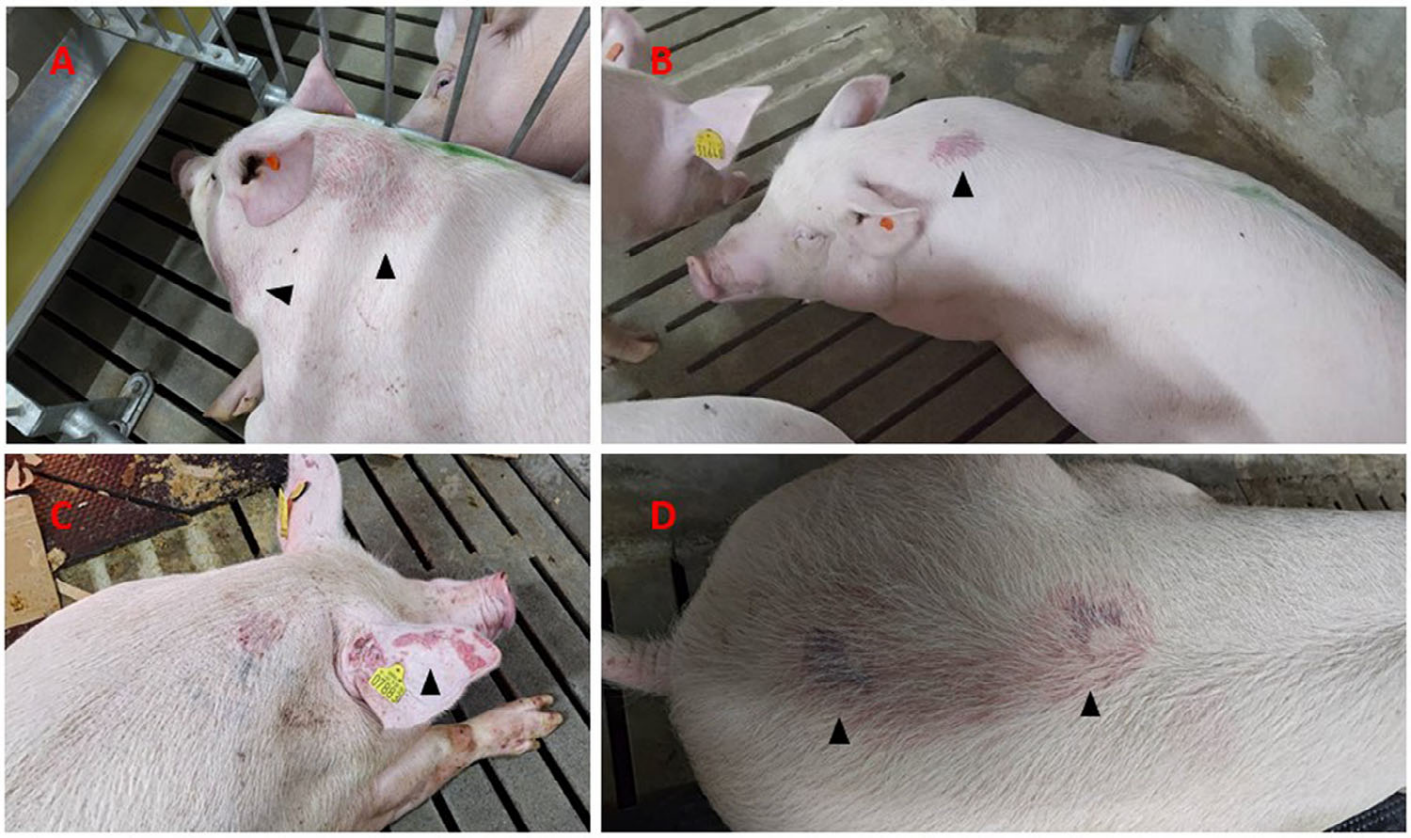
Reddish or purplish spots were observed (arrowhead), usually on the dorsal body.

**FIGURE 3:**
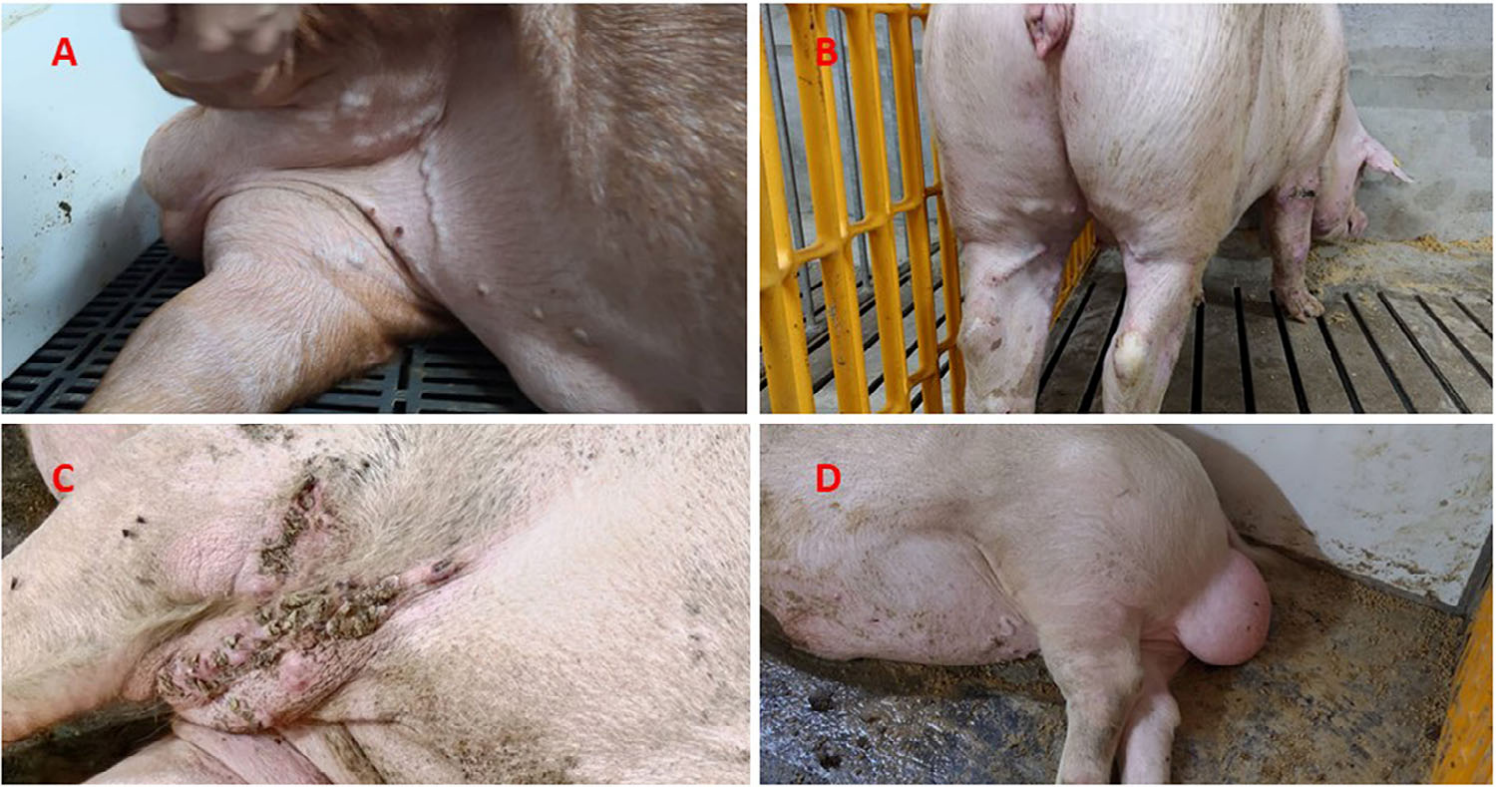
Obvious varicose veins on the abdomen and hind limbs. Additionally, Figure 2C showed decubitus sores presented along the armpit blood vessels where there was no contact with the ground.

There was 100% morbidity of lung oedema, with clinical signs showing shortness of breath with a respiratory rate exceeding 60/min, and abdominal breathing. Auscultation of the lungs revealed grunting, gurgling or wheezing sounds with breathing. In severe cases, the respiratory rate exceeded 120/min, and open‐mouth breathing was observed. Approximately 30% showed signs of hind limb weakness, making it difficult to stand up. Some displayed limb swelling, bending front hook joint, hooves caving into limbs and, in 4 pigs, paralysis. Sudden death occurred with respiratory distress, wailing and prone positioning, indicative of cardiorespiratory shock. Additionally, some young breeders experienced sudden death due to acute haemorrhagic gastric ulcers (with negative faecal occult blood tests) or haemorrhagic bowel syndrome. No fever was observed in any of the pigs. Abnormal bruising and new reddish or purplish spots were frequently observed (Figure 2) along with obvious varicose veins on the abdomen and hind limbs (Figure 3).

The elder batch showed early clinical reactions of appetite loss, depression and hind limb weakness after vaccination, prompting a blood draw for serological testing on 23 September. Over the next 4 days, clinical signs of respiration and refusal to eat became worse and continued to deterioration. Farrowing was delayed, with some sows showing no response to PGF‐2α. Post‐farrowing, sows became extremely weak, almost to the point of paralysis, refused to eat, experienced dystocia and died from debilitation. About 20% of piglets were live‐born, but all died due to lack of colostrum and milk intake. As a result, PGF‐2α was administered to other pregnant sows with clinical signs in an attempt to save more sows. Deaths started occurring on 2 October, culminating in 23 out of 34 deaths, including 1 boar and 12 sows due to cardiorespiratory shock and 10 sows dead from debilitation and refusal to eat.

In contrast, the second batch of young breeders showed loss of appetite and depression on the first day after vaccination but recovered. However, shortness of breath (50/min) was observed and did not improve. By 15 October, severe appetite loss reduced average feed intake by 40%, respiratory clinical signs and depression worsened, and instances of cardiorespiratory shock began. Deaths started on 17 October, with 13 out of 97 young breeders dying, 9 from cardiorespiratory shock, 2 from acute gastric ulcers, 1 from haemorrhagic bowel syndrome and 1 from starvation due to oesophageal atresia. Their first heat was delayed by at least 4 weeks due to severe body condition from lung oedema, although average body weight remained on schedule. Clinical signs began to improve by mid‐December. Duroc had more severe clinical signs, with 4 out of 13 dying and 2 surviving with paralysis. Landrace had a higher morbidity of hind limb weakness.

## 3 Gross Findings

Post‐mortem examination was performed on all young breeders and seven sows. Respiratory lesions, including bloated and congestive lungs, interlobular expansion and foamy liquid accumulated in the trachea, were found in all pigs. Some sudden death cases displayed bleeding points on the coronary sulcus. Case with limb weakness might have exhibited a large area of pale hind limb muscles. Most young breeders showed obvious signs of healing or recovering gastric ulcers on pars oesophagea. Two young breeders (one from the first batch who died on 25 March) experienced haemorrhagic bowel syndrome, demonstrated by thin‐walled intestines filled with clotted or unclotted blood and severe mesenteric oedema, but with no lesions suggestive of swine dysentery, salmonellosis, proliferative enteropathy or intestinal spirochetosis. Most cases showed no specific lesions on other organs, and lymph nodes were generally normal. Multiple tissue samples from the gastrointestinal tract, heart, lung, liver, spleen, kidney, lymph nodes, tonsil and brain were collected, for molecular biological examination, and simultaneously fixed in neutral‐buffered 10% formalin and routinely processed for histologic examination. Routine bacterial isolation was performed on the lung, and specific *Salmonella* isolation was conducted on the liver.

## 4 Molecular Biological and Microbiological Findings

All pathogens listed below were examined using polymerase chain reaction (PCR) or reverse transcriptase polymerase chain reaction (RT‐PCR). PRRSV and Porcine circovirus Type 2 (PCV2) were specifically identified using real‐time PCR (qPCR).

The result showed no specific pathogenic bacteria were isolated, such as *Actinobacillus pleuropneumonia, Salmonella choleraesuis, Glaesserella parasuis, Erysipelothrix rhusiopathiae* (ER) or *Streptococcus suis*. Examinations of PRV‐gE in all death cases were negative. PRRSV qPCR was examined in all death cases and in a viremia testing conducted on 23 September. The viremia testing conducted on 23 September and the examination of the first dead sow on 2 October both yielded negative results. However, PRRSV turned positive since 6 October. Other examinations for specific pathogens on specific cases, such as PCV2, ER, *Mycoplasma suis*, *Mycoplasma hyopneumoniae*, *Mycoplasma hyorhinis*, porcine parvovirus, Japanese encephalitis virus, swine influenza virus, *Brachyspira hyodysenteriae*, *Lawsonia intracellularis* or *Clostridium novyi*, were negative, except for one case each of PCV2 with a low viral load, *M. hyopneumoniae* and *M. suis*.

## 5 Histopathologic Findings

**FIGURE 4:**
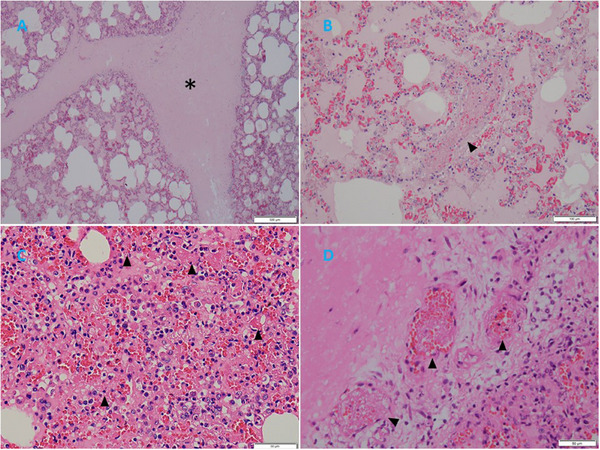
Histopathologic examination reveals pulmonary embolism. (A) Severe pulmonary oedema between pulmonary lobules (asterisk) (H&E stain, 40×). (B) Severe haemorrhage in alveolar spaces, accumulation of large amounts of proteinaceous exudate, accompanied by severe thrombus formation (arrowhead) (H&E stain, 200×). (C) Mild alveolar haemorrhage, moderate infiltration of neutrophils, accumulation of large amounts of fibrin, proteinaceous exudate, accompanied by alveolar interstitial thrombus formation (arrowhead) (H&E stain, 400×). (D) Multiple thrombus formations (arrowhead) (H&E stain, 400×).

**FIGURE 5:**
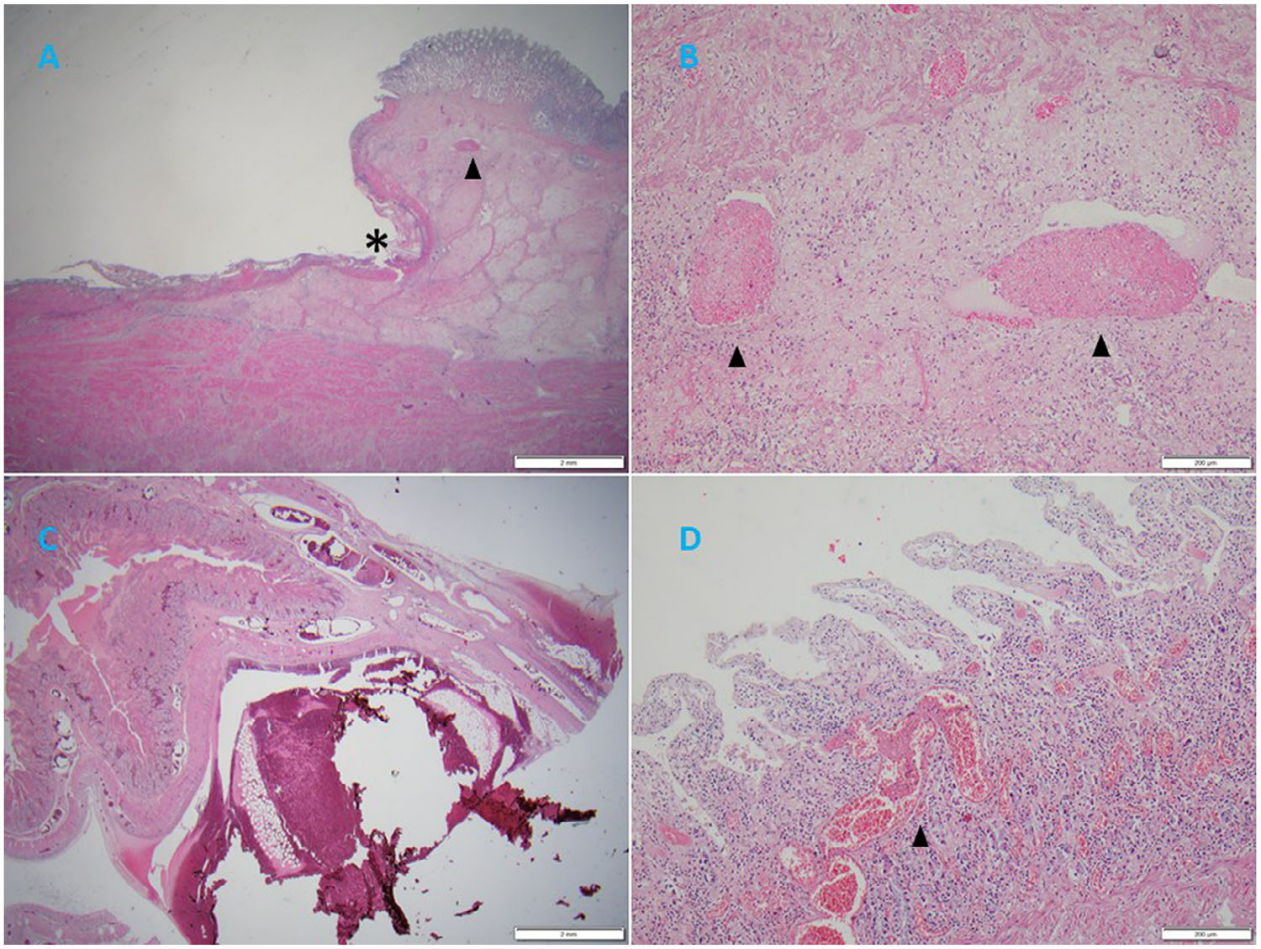
Photomicrograph of histologic sections of the stomach (A, B) and small intestine (C, D). (A) Severe gastric ulcer, deep and involving the muscular layer (asterisk), severe submucosal oedema (H&E stain, 12.5×). (B) Multiple local thrombus formations in the muscular layer (arrowhead) (H&E stain, 100×). (C) Severe haemorrhage and oedema in the serosal layer of the jejunum, dilated blood vessels, submucosal oedema (H&E stain, 12.5×). (D) Slight infiltration of plasma cells in the lamina propria of the jejunum, visible thrombus formation within blood vessels (arrowhead) (H&E stain, 100×).

Histologic examination was performed on 12 pigs. All pigs exhibited pulmonary interlobular oedema (Figure 4A). Severe acute lesions were filled with blood, cellulose and proteinaceous exudate. Cluster of lymphocytes, neophiles and some macrophages was found in the alveoli, alveolar septa and bronchi. Hyperplastic Type II pneumocytes were found only in one case, even though PRRSV was positive. Thrombi were found in 8 out of 12 cases, mostly in alveolar septa vessels or lung vessels (Figure 4). Thrombi were also found in the tonsil, lymph nodes, and liver in some cases. In addition, four cases of young breeders exhibited severe gastric ulcers at the pars oesophagea, and 3 out of 4 cases found thrombi in gastric vessels near the gastric ulcer lesion (Figure 5A,B). One case of haemorrhagic bowel syndrome also had thrombi in the small intestine vessels (Figure 5C,D). Epicardial haemorrhages were found in some sudden death cases.

## 6 Haematology and Serology Findings

For complete blood count testing by ProCyte Dx IDEXX, sampling non‐clotted blood in an EDTA tube, one blood sample was collected from a sow on 1 October, 1 day before its death, and another blood sample was collected from a gilt within 2 min after its death on 15 November. Additionally, seven samples from young breeders were collected on 3 January 2024. It is worth noting that most samples rapidly clotted, even some samples that clotted within 5 s, leading to failure in collecting them in EDTA tubes. This condition also occurred during the treatment of intravenous infusion. All samples showed thrombocytopenia (reference interval: 300–700 K/µL), with platelet counts of 272, 101, 117, 134, 199, 262, 246, 243 and 337 K/µL. Blood biochemistry testing for sodium, potassium, chloride, calcium and inorganic phosphate on 23 September and 3 January 2024, and in 2 cases, was normal.

**FIGURE 6:**
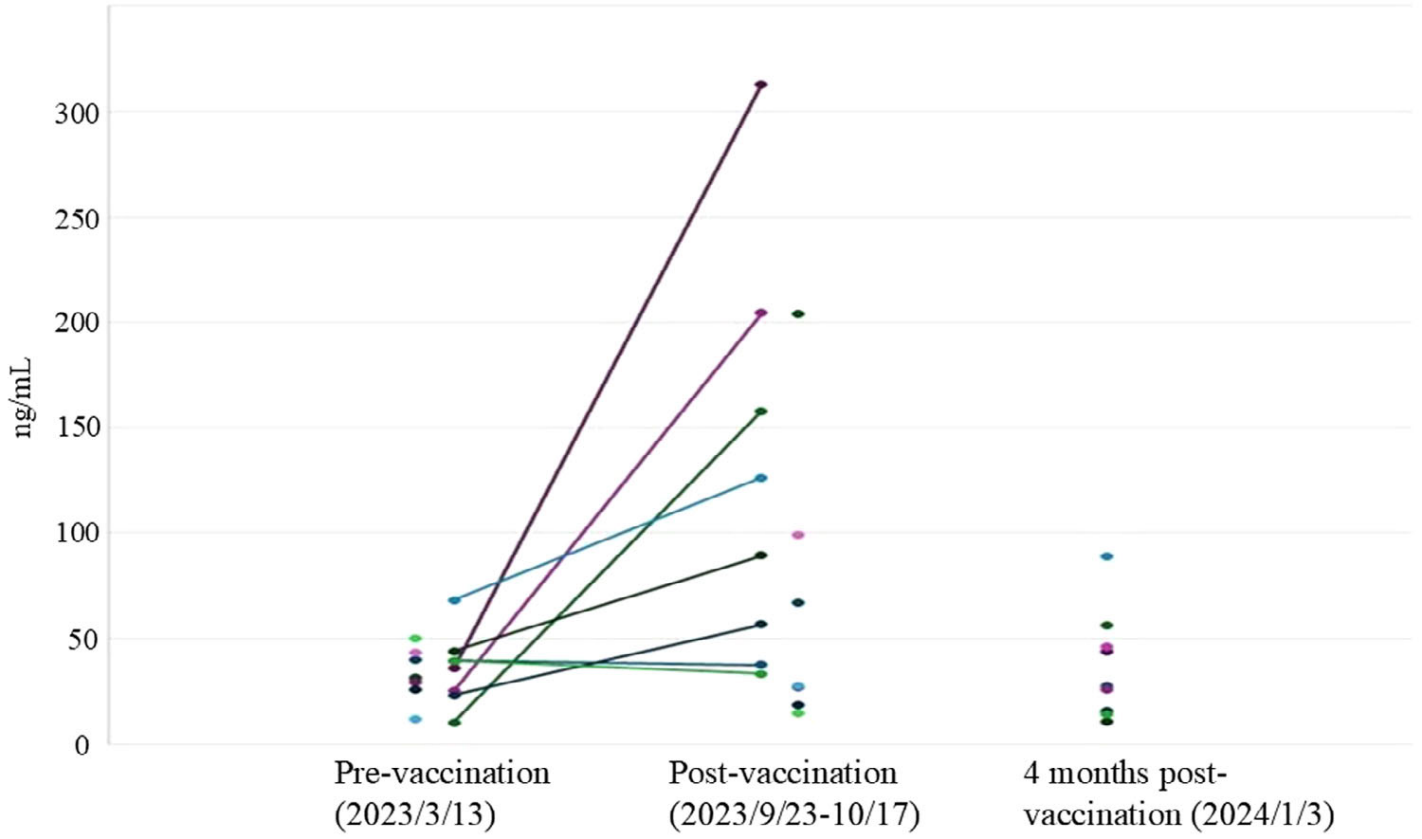
The result of anti‐heparin/PF4 ELISA testing is presented as a scatter plot, all points were individual data, grouped as follows: pre‐vaccination on March 13 (*n* = 16), post‐vaccination from 23 September to 17 October (*n* = 15), and 4 months post‐vaccination on 3 January 2024 (*n* = 9). Eight pigs, both sampling on pre‐vaccination and post‐vaccination, linked each two points show a significant rise.

For serologic testing, serum samples were collected within 2 h after death, if possible, for all cases. Group sampling was done on 13 March, 23 September and 3 January 2024. Detection of anti‐PF4 antibodies to diagnose VITT was performed using the Pig Anti‐Heparin/Platelet Factor 4 (anti‐HPF4) Antibody ELISA kit, specially made for pigs by Abbexa Ltd. The anti‐HPF4 ELISA kit can detect both anti‐PF4/heparin complex antibodies and anti‐PF4 antibodies, requiring different diagnoses between heparin‐induced thrombocytopenia (HIT) and VITT. However, since we did not use heparin on pigs, the presence of anti‐PF4/heparin complex antibodies is unlikely. Therefore, the results of the anti‐HPF4 ELISA directly reflect the value of anti‐PF4 antibodies. We compared three groups of sera: the first group was collected on 13 March, when they were pre‐vaccinated with any PRV vaccine, the second group was collected post‐vaccinations between 23 September and 17 October, and the third group was collected 4 months post‐vaccination on 3 January. The result showed that anti‐PF4 antibody levels of the post‐vaccination group (23 September–17 October) were significantly higher than the others, with a range of 14.6–313.2 ng/mL, an average of 95.9 ng/mL, and 62.5% of samples over 50 ng/mL (Figure 6).

PCV2 antibody, PRRSV antibody, PRV‐gB and PRV‐g1 antibody were detected using the ELISA kit from Biochek Ltd. Abnormal high values of PCV2 antibody were not observed. PRV‐g1 antibodies were negative, but PR‐gB antibodies showed high values with a low coefficient of variation (CV). PRRSV antibody turned positive since 6 October (Table 1).

**TABLE 1:** Results of PRRS, PCV2, PR‐gE and PR‐gB antibody testing.

		PRRS			PCV2		
Date	Sample	S/P ratio	Titre	Result	S/P ratio	Titre	Result
2023.09.23	Live sow	0.14	264	Negative	1.486	3550	Positive
2023.09.23	Live sow	0.09	162	Negative	1.053	2430	Positive
2023.09.23	Live sow	0.056	96	Negative	1.994	4906	Positive
2023.09.23	Live sow	0.055	94	Negative	2.157	5349	Positive
2023.09.23	Live sow	0.059	102	Negative	1.592	3829	Positive
2023.10.02	Dead sow	0.043	72	Negative			
2023.10.06	Dead sow	0.665	1466	Positive			
2023.10.06	Dead sow	1.055	2435	Positive			
2023.10.17	Dead young breeder	1.502	3592	Positive			
2023.11.10	Dead young breeder	0.73	1624	Positive			

		PR‐gE(g1)			PR‐gB		
Date	Sample	S/P ratio	Titre	Result	S/P ratio	Titre	Result
2023.09.23	Live sow	0.6	969	Negative	3.67	9597	Positive
2023.09.23	Live sow	0.704	800	Negative	3.352	8686	Positive
2023.09.23	Live sow	0.607	955	Negative	3.79	9943	Positive
2023.09.23	Live sow	0.687	823	Negative	4.017	10,600	Positive
2023.09.23	Live sow	0.634	907	Negative	3.213	8291	Positive
2023.10.02	Dead sow	0.561	1050	Negative	4.125	10,914	Positive
2023.10.06	Dead sow	0.691	818	Negative	4.763	12,784	Positive
2023.10.06	Dead sow	0.806	680	Negative	5	13,485	Positive
2023.10.17	Dead young breeder	0.951	557	Negative	1.404	3335	Positive
2023.11.10	Dead young breeder	0.985	534	Negative	4.888	13,154	Positive
2024.01.03	Live young breeder	0.791	695	Negative			
2024.01.03	Live young breeder	0.822	664	Negative			
2024.01.03	Live young breeder	1.091	473	Negative			
2024.01.03	Live young breeder	1.096	470	Negative			
2024.01.03	Live young breeder	0.805	681	Negative			
2024.01.03	Live young breeder	1.021	512	Negative			
2024.01.03	Live young breeder	0.939	566	Negative			
2024.01.03	Live young breeder	0.693	815	Negative			
2024.01.03	Live young breeder	0.686	825	Negative			

## 7 Case Summary

Diagnosis: (1) 100% morbidity with severe lung oedema occurring simultaneously, which has not been observed in other pathogenic diseases, even highly pathogenic PRRS (HP‐PRRS), PR or African swine fever (ASF). (2) Absence of fever and failure to isolate or detect specific pathogens. (3) Presence of reddish or purplish spots along with thrombocytopenia. (4) Significant differences in anti‐PF4 antibody levels between vaccinated and pre‐vaccinated samples (Figure 6). (5) Thrombosis observed in lung, gastric vessels, intestine vessels, liver, lymph nodes and tonsil. Following the diagnostic algorithm for patients suspected of VITT in humans (Arepally and Ortel 2021; Bussel et al. 2022; Schönborn et al. 2024): (1) clinical signs appearing 4–42 days post‐vaccination; (2) thrombocytopenia; (3) presence of thrombus in lung vessels, alveolar septa vessels, gastric vessels, and small intestine, as well as other organs, as confirmed by histopathologic examination; (4) positive result from anti‐HPF4 ELISA testing. We have summarized the case: vaccine‐induced immune thrombotic thrombocytopenia in pigs.

## 8 Treatment and Outcomes

Intravenous or subcutaneous infusion of Ringer's solution, antibiotics, NSAIDs, and high doses of dexamethasone showed limited efficacy in treating lung oedema, anorexia and paralysis. In case of severe lung oedema (respiration rate over 100/min), administering furosemide i.m. at a dosage of 120–160 mg per pig appeared to slow down the respiration rate and prevent immediate death. Products such as Ensure from Abbott Nutrition Ltd. were helpful in managing anorexia. Omeprazole p.o. at a dosage of 40 mg pig per day was used to prevent gastric ulcers but did not seem to help with acute gastric bleeding. Aspirin p.o. at a dosage of 100 mg per pig per day may have been helpful in preventing further deterioration, as most severe cases survived after treatment. However, aspirin was not administered to all pigs during the acute phase until 15 December due to concerns about potential gastric ulcer side effects. Therefore, it is unknown whether aspirin could reduce mortality and prevent sudden death if used from the beginning. While shortness of breath gradually recovered in all pigs since mid‐December, weakness of limbs, paralysis and varicose veins have not improved. The frequent appearance of reddish or purplish spots indicates that thrombocytopenia has not improved. There may be a significant risk during late pregnancy and farrowing, as splanchnic vein thromboses may flow out due to gestational hypertension or farrowing pressure, causing severe damage to the lungs, heart and other organs. This speculation is based on the observation that at least 20% of sows from the first batch exhibited moderate weakness of hind limbs, front hock joint bending, loss of appetite, depression and even paralysis in August and September before vaccination. At that time, the incorrect diagnosis was osteochondrosis (OCD) or calcium/phosphorus imbalance and heat stress. Thromboses were found to be irreversible, and the damage from post‐thrombotic syndrome (PTS) and pulmonary embolism (PE) was also challenging to recover from, such as decreased respiratory function, infertility due to damage to the uterus, lameness and varicose veins. Therefore, our approach is to continuously administer aspirin p.o. to minimize the damage from PTS and PE when deep splanchnic vein thromboses flow out again.

## 9 Comments

VITT is a novel syndrome that unexpectedly emerged along with the global effort to fight COVID‐19 in humans. It was reported in 2021. Rarely, healthy individuals developed complications of thrombocytopenia and thrombosis in atypical locations (cerebral and/or splanchnic veins) within weeks of receiving the adenoviral‐based vector vaccines (AstraZeneca and Janssen/Johnson & Johnson). VITT is a clinically distinctive syndrome that exhibits (1) a propensity for cerebral and/or splanchnic vein thromboses, (2) laboratory features showing consumptive coagulopathy in association with anti‐PF4 seropositivity and (3) poor outcomes (Arepally and Ortel 2021). Clinical signs may be performed: persistent and severe headache, focal neurological symptoms (including blurred or double vision), shortness of breath, chest, back, or abdominal pain, unusual bleeding, bruising, petechiae, or blood blisters, swelling and redness in a limb, or pallor and coldness in a limb. VITT seems to occur between 4 and 42 days post‐vaccination (Pai et al. 2021; Bussel et al. 2022). Diagnosis is based on low platelets, high D‐dimer and clinical signs starting 4–42 days post‐vaccination (Bussel et al. 2022). The confirmatory diagnosis of VITT is made using tests that are also used for HIT, detecting anti‐heparin/PF4 antibodies by ELISA (Pai et al. 2021). Rapid initiation of high‐dose intravenous immune globulin (IVIG) treatment is the most useful approach, often paired with non‐heparin anticoagulation. Heparin, warfarin, aspirin and corticosteroids should be avoided as treatment or prophylaxis, as they rarely prove useful and may enhance the risk of clotting or bleeding (Bussel et al. 2022).

In relation to PTS caused by VITT, we found key evidence suggesting that PTS may be the underlying cause of gastric ulceration, haemorrhagic bowel syndrome and OCD/leg weakness syndrome or may have a significant association with them. We deduced that gastric ulcers were part of PTS based on the following observation: (1) abnormally high morbidity and mortality in our young breeders; (2) thrombosis found in gastric vessels in three out of four cases with severe gastric ulcer at pars oesophagea (Figure 5A,B) and (3) severe bleeding cases that resulted in sudden death without tarry faeces, leading us to consider gastro‐oesophageal varices and bleeding in humans caused by circulatory disorders. Regarding PTS and haemorrhagic bowel syndrome, we observed the following: (1) moderate lung oedema was observed through histopathologic examination in the first case on 25 March; (2) thrombus appeared in the vessels of the small intestine in the second case on 26 November (Figure 5C,D) and (3) severe oedema of the mesentery was found in both cases, which might be caused by increased hydrostatic pressure of PTS or reduced fibrinogen due to activated coagulation. Concerning OCD, all the clinical characteristics closely matched those of our breeders (Schwartz 2023), but with abnormally high morbidity. We observed five cases of paralysis and one severe case. As well as several mild to moderate cases with hind limb weakness, hind foot tapping, bending of the front hock joint, a tendency to slip, and difficulty standing for extended periods of time. Upon reviewing the cases, we found that hind limb weakness and clinical signs of OCD were first observed within 7–21 days post‐vaccination and gradually worsened, with at least 20% of our breeders exhibiting clinical signs by the end. Therefore, we consider a potential link between PTS and OCD. It is possible that PTS is causing the loss of vascular supply to the growth plates, leading to the development of OCD. Additionally, the reasons for gastric ulceration, haemorrhagic bowel syndrome, and OCD were not fully confirmed, but they commonly occur at 3–6 months of age (Thomson and Robert 2012; Burrough 2021; Schwartz 2023), when after the first PRV vaccination was regularly administered.

VITT typically occurs between 4 and 42 days after ‘the first vaccination’ in humans, which corresponds to our situation. Obvious clinical signs were observed in the second batch 2–3 weeks after vaccination. Additionally, in retrospect, the first batch also presented clinical signs after previous vaccinations. This included one pig succumbing to haemorrhagic bowel syndrome, another developing a severe gastric ulcer, a boar experiencing paralysis, and 50% morbidity with shortness of breath, which was misdiagnosed as heat stress (Figure 1). However, the prompt reaction seen in the first batch of sows and boars after 22 September could be attributed to the rapid generation of pathogenic anti‐PF4 antibodies following repeated stimulation.

We observed that, if under the same conditions with one vaccination, the second batch exhibited more severe clinical signs than the first batch. We suspect that factors such as PRRSV infection, heat stress, and immunodepression may have enhanced and prolonged the effects of the PRV live vaccine. This indirectly suggests why VITT might not have been identified earlier, as most attention was focused on PRRSV infection when severe clinical signs occurred. In fact, simple PRRSV infection should not cause such high mortality.

Although the interpretation criteria for anti‐PF4 antibodies have not been established in pigs, there was a significant increase between the pre‐vaccination samples and the post‐vaccination samples (23 September–17 October). This included eight pigs that were sampled at both times and aligned with each other (Figure 6). The pre‐vaccination samples were tested with previous lots of ELISA kits, and the results matched the SPF pig reference from Abbexa Ltd. (data not shown). Therefore, the anti‐HPF4 ELISA test to confirm that VITT also works in pigs. Regarding the follow‐up of anti‐PF4 antibodies, in humans, these antibodies can be detected for a long time. However, the functional test and ‘platelet‐activating’ anti‐PF4 antibodies typically turn negative within 15 weeks (Schönborn et al. 2022). In our cases, the anti‐PF4 antibodies turned negative at 16 weeks post‐vaccination, which differs from the human situation. I speculate that all anti‐PF4 antibodies in pigs may be ‘platelet‐activating’ anti‐PF4 antibodies, which combine with platelets to form thrombi. Therefore, not only were the 16‐week post‐vaccination samples low or negative for anti‐PF4 antibodies, but some acute VITT samples were as well.

In Taiwan, a low survival rate of imported Danish pure breeders, below 80% before insemination, has been reported. There have also been cases of over 20% mortality due to gastric ulcers. We often attribute these failures to pathogenic stress, heat stress, environmental factors, improper diet, moving stress and genetic issues. However, it is important to consider VITT and PTS in these situations. Imported pure breeders in Taiwan must be PRV‐free, and some countries have achieved PRV purification without using any vaccines, such as Denmark and Britain. The lack of adaptation to the vaccine or virus may explain why our breeders experience severe VITT/PTS, while similar syndromes have not been reported in other Taiwanese pigs.

Furthermore, the adjuvant used in Zoetis PR‐VAC PLUS may enhance the effects of the live vaccine and stimulate the production of higher levels of pathogenic antibodies, thus causing more severe VITT.

## 10 Follow‐Up and Possible Conclusions

Follow‐up on 11 May 2024: Our surviving sows have started farrowing, and similar to the outcomes observed in humans, the prognosis is very poor and lifelong due to the incurable thrombosis leading to infertility, leg weakness or paralysis, decreased lung function and the presence of multiple abnormal necrotic cysts. Additionally, when the pregnancy reaches 108–112 days, sows develop antibodies for colostrum. The production of anti‐PF4 antibodies, which are associated with VITT, occurs again at this stage. This results in sudden severe lung oedema, stillbirths, dystocia, delayed farrowing, agalactia, loss of appetite, leg weakness, paralysis and death, with 90% morbidity and over 10% mortality despite treatment (Figure 1). Severe clinical signs were very similar to those observed in cases on September–October 2023. However, PRRSV qPCR examinations were negative this time. Therefore, this situation confirms that pathogenic anti‐PF4 antibodies may directly lead to TTS (instead of using VITT, as there was no vaccination this time). This also explains why the mortality in the first batch was so high, matching the two situations of repeated vaccination and post‐term pregnancy.

VITT in pigs, not previously reported, could be attributed to several factors: (1) Differences in diagnosing pigs compared to humans, especially considering that VITT associated with COVID‐19 vaccines has only recently been identified. (2) Challenges in linking clinical signs such as pulmonary oedema, OCD/leg weakness, gastric ulcers and haemorrhagic bowel syndrome to thrombosis. (3) Delayed onset of clinical signs 4–42 days post‐vaccination, unlike common vaccine side effects. In addition, it could even lead to TTS when sows near farrowing produce pathogenic antibodies again. (4) Similar to the urgent medical evaluation for VITT in humans, where clinical signs were varied and illogical (Bussel et al. 2022), as seen in our first batch with previous vaccinations, the clinical signs ranged from mild to moderate with low mortality. It was challenging to infer VITT without a comprehensive investigation to detect thrombosis. Therefore, the anti‐HPF4 ELISA test emerged as the definitive diagnostic method. (5) Difficulty in diagnosing the condition due to the presence of common pathogens like PRRSV and PCV2. Due to the challenges in diagnosis and the presence of various mild‐to‐severe clinical signs, VITT may have occurred before but went unnoticed. In Taiwan, purebred Danish pigs experience over 20% mortality before the first farrowing, with performance significantly differing from that in Denmark. This difference is often attributed to diseases and climate issues. (6) Genetic factors may play a crucial role. Danish Genetics bred pigs with highly centralized and stable genes. We observed that the same strain showed similar adverse reactions. For example, there was a high mortality rate among 10 out of 12 Danish Duroc pigs that either died or were culled, exhibiting more severe clinical signs. Leg weakness appeared more often in Landrace pigs. Additionally, they bred high‐immunity pigs, as observed with abnormally high PR‐gB antibody levels even after just one vaccination dose. Therefore, centralized genetics may be associated with 100% morbidity of VITT, and high immunity means producing more pathogenic anti‐PF4 antibodies, leading to more severe adverse reactions. In this way, pigs with strong immunity may appear more susceptible to VITT. However, the potential problem of VITT could not be identified in Denmark because no PR vaccine is used due to the successful eradication of PRV without vaccines. Additionally, as previously mentioned, VITT is difficult to diagnose and notice. Conversely, the concerning hypothesis is that we might unintentionally filter out pigs with stronger immunity through PRV vaccination.

Understanding the occurrence of VITT could be based on virus strains, pig genes or other reasons in further research. We should focus not only on the presence of pathogenic anti‐heparin/PF4 antibodies but also on the expression of FcγRIIA or other Fc receptors and their interactions. In humans, FcγRIIA is expressed on platelets, monocytes and neutrophils, cross‐links with the anti‐heparin/PF4 antibody, triggering procoagulant cellular responses that lead to the development of a profound hypercoagulable state (Arepally and Padmanabhan 2021). However, very little is known about the Fc‐mediated functions of porcine IgG subclasses (Paudyal et al. 2022).

Is there a possibility of reversion to the virulence of the PRV vaccine strain virus? Based on our analysis, we believe not. Firstly, the antibody response to the PRV virulence gene, gE, remains negative. Secondly, PCR testing for the PRV vaccine virus has shown negative results. However, the memory B cells responsible for producing pathogenic anti‐PF4 antibodies and thrombosis may still be present in the pigs, as the recurrence of clinical signs occurs specifically around Days 108–112 of pregnancy, rather than over a period typical of common infectious diseases.

VITT is a vaccine safety signal associated with the PRV live vaccine that requires reassessment. It is possible that VITT not only affects Danish purebred pigs but also impacts other pigs, which may be adapted to the PRV vaccine with fewer or no adverse reactions. It is important to ascertain the pathogenesis of VITT if PRV eradication is still our ultimate goal. Additionally, this model of inducing VITT can help us explore the condition further and assist the vaccine industry in both human and veterinary medicine.

## Author Contributions

**Chien‐Cheng Chen**: writing–original draft.

## Ethics Statement

The author has nothing to report.

## Conflicts of Interest

The author declares no conflicts of interest.

### Peer Review

The peer review history for this article is available at https://www.webofscience.com/api/gateway/wos/peer‐review/10.1002/vms3.70222.

## Data Availability

The data that support the findings of this study are available from the corresponding author upon reasonable request.
